# Novel *RGAG1-BCOR* gene fusion revealed in a somatic soft tissue sarcoma with a long follow-up

**DOI:** 10.1007/s00428-021-03160-z

**Published:** 2021-07-31

**Authors:** Mauro Vasella, Ulrich Wagner, Christine Fritz, Kati Seidl, Luca Giudici, Gerhard Ulrich Exner, Holger Moch, Peter Johannes Wild, Beata Bode-Lesniewska

**Affiliations:** 1 Department of Plastic Surgery and Hand Surgery, University Hospital Zurich, Rämistrasse 100, 8091 Zurich, Switzerland; 2 Institute of Pathology and Molecular Pathology, University Hospital Zurich, Zurich, Switzerland; 3 Orthopaedie Zentrum Zuerich, Ozz, Zurich, Switzerland; 4 Senckenberg Institute of Pathology, Goethe University Frankfurt, Frankfurt, Germany; 5 Present Address: Pathology Institute Enge and University of Zurich, Zurich, Switzerland

**Keywords:** Sarcoma, Ewing-like, Next-generation sequencing, BCOR, RGAG1

## Abstract

*BCOR*-rearranged sarcomas are rare and belong to the Ewing-like sarcomas (ELS). Their morphology and histopathological features make the diagnosis challenging. We present a case, initially diagnosed as an unusual extraskeletal myxoid chondrosarcoma (EMC). A 54-year-old male patient developed an asymptomatic swelling of the lower leg. Imaging showed a 9.5-cm large intramuscular soft tissue mass. Due to its morphological and immunohistochemical profile on biopsy, it was initially diagnosed as an EMC. The patient was treated by complete resection and adjuvant radiotherapy and remained free of tumor at 7 years follow-up. Using next-generation sequencing (NGS), we retrospectively identified *RGAG1-BCOR* gene fusion (confirmed by RT-PCR), which has not been described in somatic soft tissue tumors so far. This finding broadens the spectrum of partner genes in the *BCOR*-rearranged sarcomas in a tumor with a well-documented, long clinical follow-up.

**Supplementary Information:**

The online version contains supplementary material available at 10.1007/s00428-021-03160-z.

## Introduction

*BCOR*-rearranged sarcomas (BRS) represent a rare type of small round cells sarcoma and are listed separately as “sarcomas with BCOR genetic alteration” in the latest WHO classification [1]. BRS were first described by Pierron et al. and lack *EWSR1* rearrangement [2, 3]. *BCOR* stands for *BCL-6* *corepressor*, which interacts with the *BCL-6* gene. *BCL-6* regulates immune responses and can stimulate or inhibit apoptosis [4]. Moreover, it has been previously shown to play a role in the pathogenesis of solid and hematologic malignancies [5–7].

BRS usually emerges in bones of the lower limbs [3, 5, 8–10]. Men are more prone to develop BRS in their second decade [3, 5, 8–10]. Five-year overall survival rates are around 75%, similar to the overall survival rates of Ewing sarcoma (ES) [2, 3, 8]. Clinically, BRS usually presents as a painful mass [8]. Treatment is also similar to ES, with complete resection being the most crucial one [9].

Histologically, BRS consists of uniform ovoid or round cells with spindled pattern and myxoid stroma [3, 9].

Immunohistochemistry may show positivity for CD99, BCL2, SATB2, BCOR, and TLE1, making its diagnosis challenging, with several differential diagnoses, including synovial sarcoma or chondrosarcoma variants [3, 3, 9, 10].

The most common rearrangement found in BRS is a *BCOR-CCNB3* gene fusion. Due to the emergence of next-generation sequencing (NGS) as a diagnostic tool, other translocations have been identified in BRS including *BCOR-MAML3*, *ZC3H7B-BCOR*, *KMT2D-BCOR*, and *ZC3H7B-BCOR* [11].

Here, we present a unique first case of somatic soft tissue sarcoma with a *RGAG1-BCOR* gene fusion and clinical long follow-up.

## Materials and methods

### Case report

**Fig. 1:**
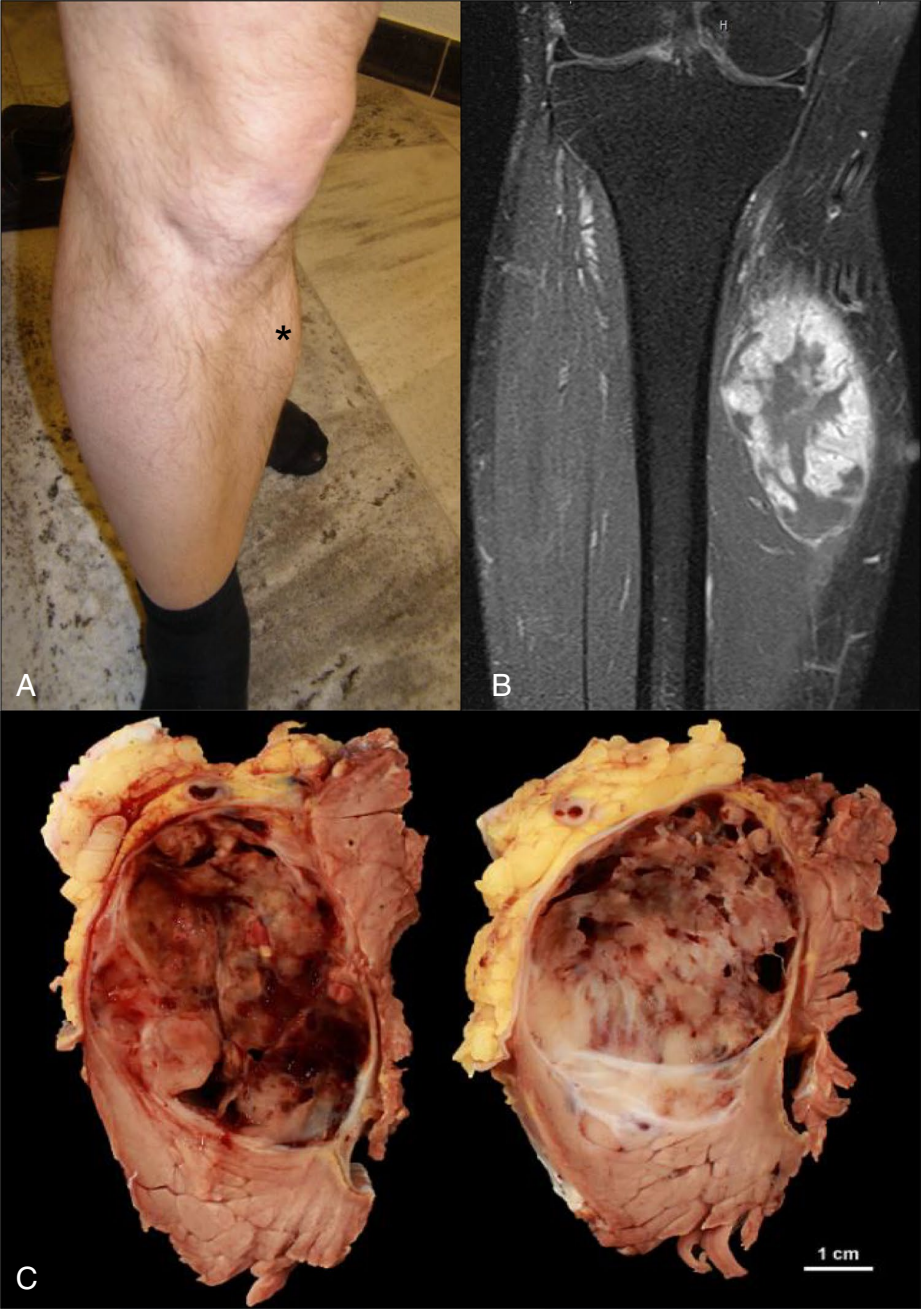
Presentation of the tumor. Clinical presentation of the tumor in form of a medial swelling (**A**, asterisk). MRI exam in T1 sequence and coronal plain showing no infiltration of the tibia (**B**). Macroscopic presentation of the resected tumor (**C**)

A 54-year-old man presented with a large intramuscular, painless swelling in his lower leg (Fig. 1A). Ultrasound and magnetic resonance imaging (Fig. 1B) revealed a well-demarcated intramuscular tumor (95 × 50 × 40 mm) that was highly indicative of sarcoma. Core biopsy showed a myxoid mesenchymal neoplasia and staging work-up excluded metastatic disease. Complete resection with clear resection margins was performed (Fig. 1C). Reverse transcription-polymerase chain reaction (RT-PCR) and fluorescence in situ hybridization (FISH) were performed and excluded the diagnosis of a myxoid/round cell liposarcoma. NGS was not available as a routine diagnostic tool at this time, and therefore, the diagnosis of exclusion was an unusual cellular EMC. However, we were not able to confirm this diagnosis on the molecular level, since a test for the detection of *NR4A3* gene rearrangements was not available. The patient received adjuvant radiotherapy (cumulative 60 Gy), but no chemotherapy. Five years later upon availability, NGS analysis of the tumor was performed detecting a *RGAG1-BCOR* gene fusion. The patient was still under complete remission after 48 months of follow-up. However, more recently, the patient has been diagnosed with a *NRAS* gene–mutated melanoma by another institution originating in the right gluteal region with lymph node metastasis, without relation to the sarcoma. He received immunotherapy for melanoma. At 87 months of follow-up since sarcoma diagnosis, there are no signs of recurrence or metastases of both tumors.

### Ethics

The patient signed and agreed to the general patient consent according to ethical standards of the University Hospital Zurich, allowing the use of all tissue, cells, and images in this report.

### Immunohistochemistry

Staining was performed by an automated IHC system on formalin-fixed and paraffin-embedded tissue of representative areas of the tumor cut into 2-μm-thick sections according to the protocol used in our institute [12]. Samples were loaded on the BenchMark Ultra (Ventana) or the Bond-Max system (Leica). The following antibodies were used: S100 (Dako), synaptophysin (Novocastra), SOX9 (Millipore), cytokeratin (Dako), CD99 (Novocastra), CD34 (Ventana/Roche), smooth muscle actin (Sigma), calponin (Dako), desmin (Dako), HMB45 (Dako), p63 (Ventana / Roche), caldesmon (Dako), epithelial membrane antigen (Dako), Ki-67 (Ventana/Roche), WT1 (Ventana/Roche), BCOR (Santa Cruz Biotechnology), and CCNB3 (SIGMA Chemical Company).

### Reverse transcription-polymerase chain reaction

RNA was extracted from paraffin-embedded tissue as previously described [13]. For the detection of fusions typically found in liposarcoma, reverse transcription of the RNA into cDNA and its amplification were performed using the OneStep RT-PCR kit (Qiagen), the GeneAmp PCR system 9700 (Applied Biosystems), and the following primers: *EWSR1-DDIT3* type 1 (5′-TCCTACAGCCAAGCTCCAAGTC-3′, -5′-CCGAAGGAGAAAGGCAATGACTCAG-3′), *EWSR1-DDIT3* type 2 (5,-GAC CCATGGATGAAGGACC-3′, 5′CCGAAGGAGAAAGGCAATGACTCAG-3′), *FUS/TLS-DDIT3* type 1 (5′-GGAAGTGACCGTGGTGGCTT-3 ′, 5′-CCGAAGGAGAAAGGCAATGACTCAG-3′) and *FUS/TLS-DDIT3* type 2 (5′-GCAGAACCAGTACAACAGCAGCAGTG-3′, 5′-CCGAAGGAGAAAGGCAATGACTCAG-3′). RT-PCR was performed as previously described by Bode et al. [14].

For the confirmation of the *RGAG1-BCOR* fusion by RT-PCR, the following primers were used: 5′-GCTCTGTGGAGGAAGAGATG-3′ and 5′-GGATTCTCTTCCCTCAGTTC-3′. The reverse transcription was done for 30 min at 50 °C, followed by a PCR activation step for 15 min at 95 °C and 40 cycles consisting of 1 min at 94 °C, 1 min at 55 °C, and 1 min at 72 °C. RT-PCR products were analyzed by 2% MetaPhor agarose gel electrophoresis. Bands were excised and subsequently purified with a MinElute Gel Extraction Kit (Qiagen). Direct sequencing of the PCR products was done on an ABI 3130xl Sequencer using the Big Dye Terminator v1.1 Cycle Sequencing Kit (all from Applied Biosystems).

### Fluorescence in situ hybridization

The probe kit used to detect the *DDTI3*(CHOP) gene on chromosome 12q13 was the LSI *DDTI3* kit (Abbott) containing a 700-kb probe labeled with SpectrumOrange (centromeric) and a 663-kb probe with SpectrumGreen (telomeric). Detection of the *EWSR1* gene on chromosome 22q12 was performed using LSI *EWS* (Abbott) consisting of a 497-kb probe labeled with SpectrumOrange (centromeric) and a 1100-kb probe with SpectrumGreen (telomeric). Both kits were used according to the protocol of Abbott Molecular. 50 cells were examined and the cut-off for positive rearrangement was set at 25% of cells presenting split signals. The protocol corresponded to Wolpert et al. [12].

### RNA sequencing

RNA isolation was performed using the Maxwell 16 LEV RNA FFPE Purification Kit (Promega Corporation) and RNA quality was assessed on a Bioanalyzer using the RNA 6000 Pico Kit (Agilent) according to the manufacturer’s instructions. NGS Libraries were prepared using the “TruSight RNA Pan-Cancer Panel” (Illumina, Inc). Quality and quantity were determined with the DNA 1000 Kit and Bioanalyzer (Agilent). Libraries were sequenced on the MiSeq platform (Illumina, Inc). The RNA fragments were sequenced using the MiSeq system (Illumina, Inc., USA).

### Analysis of RNA sequencing results with FusionMap

The FusionMap software was used to detect RNA fusion reads according to Ge et al. [15].

## Results

### Pathologic findings

Resected specimen contained a poorly demarcated, lobulated tumor measuring 5.7 × 4.1 × 10.5 cm, white-greyish with focal hemorrhages (Fig. 1C).

**Fig. 2:**
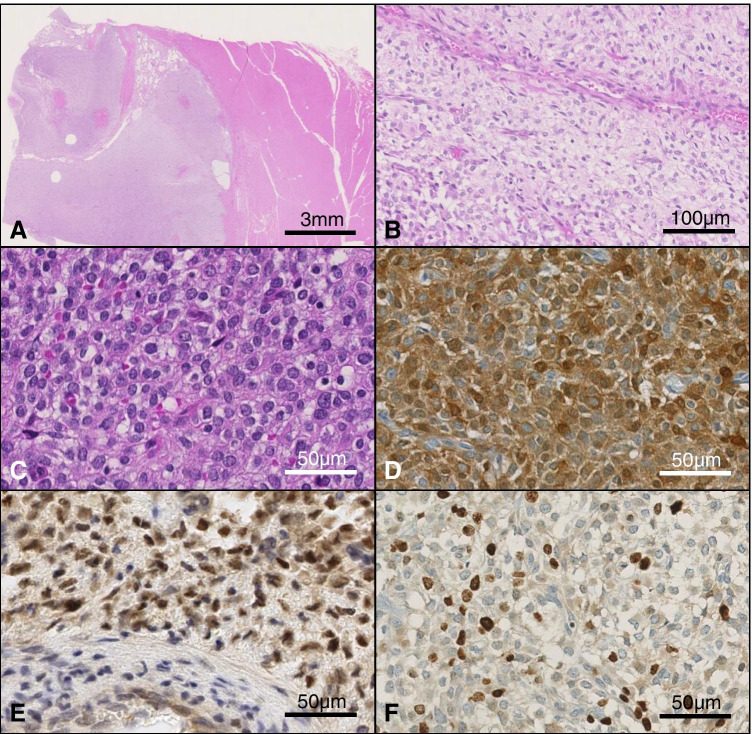
Microscopic presentation of the resected tumor. Staining with hematoxylin and eosin (H/E) showing lobulated pattern (**A**), H/E staining demonstrating myxoid areas (**B**), H/E with monomorphic cells containing narrow cytoplasm (**C**), positive S100 (**D**), strongly positive *BCOR* (**E**), and *MIB1* with a proliferation rate up to 40% (**F**); magnification × 200

Histologically, the tumor presented as polylobulated and had myxoid stroma with some hyalinized areas. It showed multinodulary invasive growth into the surrounding tissue. The cells were monomorphic with relatively narrow cytoplasm and monomorphic nuclei with finely stippled chromatin (Fig. 2A). The tumor was rich in mitotic Figures (21/10 HPF).

The initial immunoprofile was rather unspecific with focal positivity for S100, synaptophysin and SOX9 (Fig. 2B). Reactions for pan-cytokeratin, CD99, CD34, SMA, calponin, desmin, HMB45, p63, caldesmon, and EMA were negative. The proliferation index of *MIB1* was heterogeneous of up to 40% (Fig. 2D). Staining with antibodies against *BCOR* and *CCNB3* was carried out after NGS analysis, showing strong nuclear expression of the BCOR protein (Fig. 2C), while CCNB3 reaction was negative.

### Fluorescence in situ hybridization

No rearrangement of the *DDTI3*(*CHOP*) gene was detected, excluding differential diagnosis of liposarcoma. There was no rearrangement of the *EWSR1* gene.

### Next-generation sequencing and RT-PCR followed by Sanger sequencing

The initially performed RT-PCR showed no t(12;16) and (12;22)(*FUS-DDIT3* and *EWSR1-DDIT3)* specific fusion transcripts.

Due to unusual histopathologic features of the tumor, the NGS has been performed upon availability retrospectively. Using the FusionMap, we were able to detect three fusions with *RGAG1* and *BCOR* genes as fusion partners. For the first two fusions, *BCOR* was found in 5′ position and RGAG1 in 3′ position. The breakpoints of the first fusion sequence (Suppl. Figure 1) were found in exon 2 of *BCOR* and exon 2 of RGAG1, whereas the breakpoints of the second fusion sequence (Suppl. Figure 2) were found in exons 2 of *BCOR* and in the promoter region of *RGAG1*. For both of these fusion products, their potential open reading frames showed a stop codon within the first six amino acid positions from the fusion junction, making the potential protein products most likely non-functional.

**Fig. 3:**
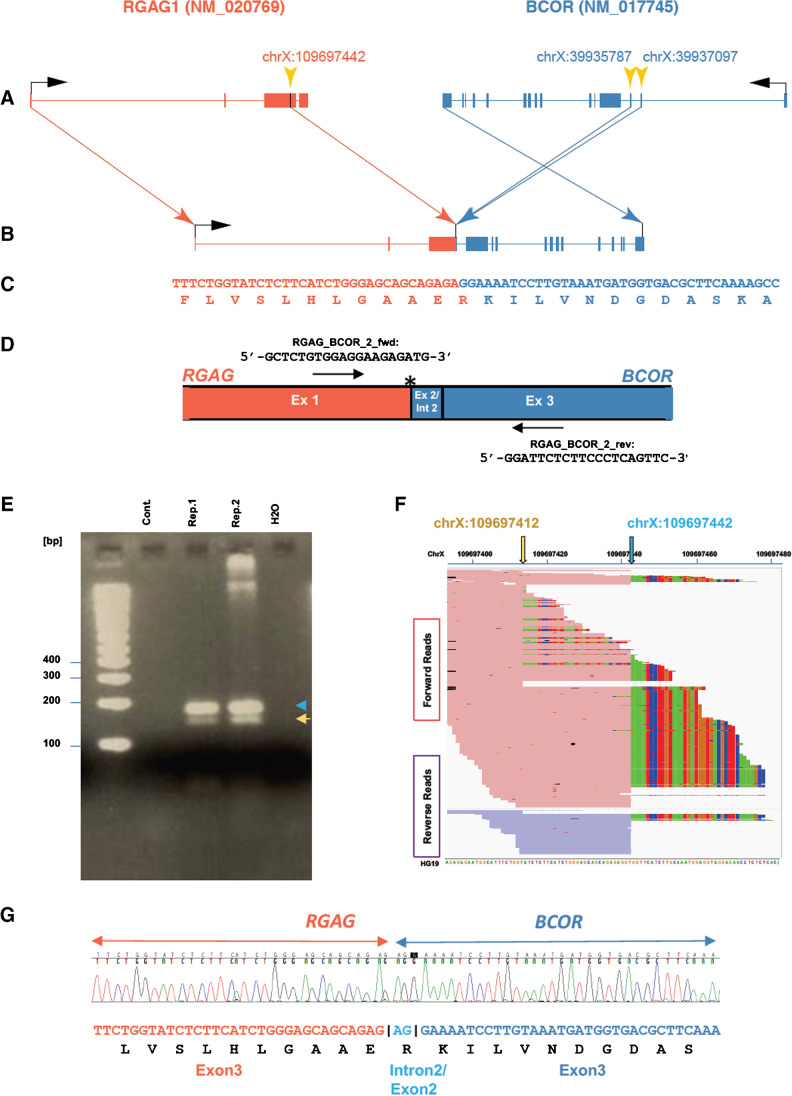
NGS. A novel *RGAG1-BCOR* gene fusion transcript predicted to be functional. Full intron–exon-structure of the two fused genes is shown in (**A**) and the respective breakpoint positions are indicated by yellow arrows. As the exact location of the breakpoint in the *BCOR* gene is ambiguous, we indicated the location of two possible breakpoints. (**B**) shows the intron–exon-structure of the resulting fusion transcript. The consensus sequence of all reads spanning the junction is shown in the upper row of (**C**). The lower row shows the predicted translation product. The RT-PCR-strategy for the detection of this gene fusion is given in (**D)**. The RT-PCR fragments were separated on an agarose gel in duplicate samples (**E**). The fragment corresponding to the fusion described in panel A is indicated by a blue arrow, whereas a second, slightly smaller fragment corresponding to the fusion product shown in Supp. Fig. 3 is marked by a yellow arrow. Sequencing reads aligned by FusionMap confirm the existence of two breakpoints (indicated by blue and a yellow arrow) in the *RGAG1* gene with a distance of 30 bp of each other (**F**). The colorfully striped moieties  of the sequencing reads indicate soft-clipped reads, which represent the part of the fusion read that aligns to the region of the *BCOR* gene (as indicated in **A**) and not to *RGAG1*. The Sanger sequencing results with electropherogram, cDNA sequence in red and blue letters, and corresponding amino acid sequence in black are shown in (**G**). The two nucleotides with ambiguous alignment to the reference sequence are marked by lighter blue color

The third sequence reported by the FusionMap represented a fusion of *RGAG1* in 5′ position and *BCOR* in 3′ position (Fig. 3A and B). The breakpoint in the *RGAG1* gene was found in exon 3, whereas the alignment by FusionMap indicated the position of the breakpoint in the *BCOR* gene at the 3′-end of intron 2 (two base pairs upstream of the 5′-end of exon 3). Alternatively, the breakpoint in the *BCOR* gene could be two base pairs upstream of the 3′-end of exon 2. The detection of this fusion was supported by 61 paired reads (i.e., one read in the *BCOR* and the corresponding paired reads in the *RGAG1* gene) and 268 junction-spanning reads. Using RT-PCR (Fig. 3D) and Sanger sequencing, we could confirm the existence of this gene fusion (Fig. 3E and G). However, the exact location of the breakpoint in *BCOR* remained ambiguous as we analyzed RNA and not DNA. Nevertheless, we predict the expected fusion product to be in frame for both *RGAG1* and *BCOR* (Fig. 3C and G). Furthermore, RT-PCR revealed the existence of a second *RGAG1-BCOR*-fusion that is 30 bp shorter than the first *RGAG1-BCOR*-fusion. We were able to confirm this finding by visual inspection of the sequencing reads obtained by the Pan-Cancer panel (Fig. 3F). Also, for this fusion product, we predict a stop codon within 6 AA from the fusion point (Suppl. Figure 3).

## Discussion

At the time of a primary diagnosis subtyping of the entity of this unusual soft tissue tumor was difficult, as it did not fit in any of the defined subtype categories. The diagnosis of exclusion was EMC. The better understanding of pathogenesis in soft tissue tumors combined with NGS as a diagnostic tool led to the detection of four fusions of the *RGAG1* and the *BCOR* gene, with probably only one of these fusions being functional (Fig. 3).

BRS are separately classified within the ELS group in the new WHO classification [1]. The genetic aberrations of BRS comprise either BCOR gene rearrangements or internal tandem duplications [7]. The *RGAG1-BCOR* gene fusion detected in our case has not been reported before in the context of a somatic soft tissue sarcoma. However, there is one case of a uterine sarcoma with *RGAG1-BCOR* fusion described in the literature [16]. *RGAG1* is located on the X-chromosome (Xq23), indicating a paracentric inversion similar to the rearrangement of the *CCNB3* gene. The function of *RGAG1* remains unknown [17].

BRS have been characterized by a nuclear upregulation of the BCOR protein. Retrospective staining using a BCOR antibody also showed nuclear upregulation of *BCOR* in our case, while CCNB3 was negative. Further immunohistochemical analyses revealed similar results as reported in the literature with unspecific staining of CD99, BCL2, SATB2, BCOR, and TLE1 as well as negativity for cytokeratins, desmin, and CD34 [3]. The immunohistochemical profile of the tumor is similar to the profile for BRS. There were areas of solid sheaths of monomorphic, middle-sized cells with less cellular areas with trabecular growth, myxoid background, and a network of thick-walled blood vessels. No osteoid production was observed, differentiating the tumor from an ossifying fibromyxoid tumor, which may show microscopically similar areas and express S100 immunohistochemically.

The *BCOR-CCNB3* tumors seem to have a similar overall prognosis to ES and respond to the same treatment. No local recurrence or metastases were detected in our patient within the 7-year follow-up despite the fact that he had not received chemotherapy.

In conclusion, we present a case of BRS with a novel fusion type *RGAG1-BCOR* which was primarily diagnosed as EMC. Novel high throughput molecular methods help to detect unexpected and unusual molecular aberrations in mesenchymal tumors allowing a better understanding of their pathogenesis.

## Supplementary Information

Below is the link to the electronic supplementary material.

Supplementary file1 (PPTX 109 KB)

Supplementary file2 (PPTX 101 KB)

Supplementary file3 (PPTX 118 KB)

## Data Availability

All data, materials, and diagnostics have been collected and used in the University Hospital Zurich.

## Code availability

Software applications have been declared in the “Materials and methods” section.
