# Supplementary figures and images for: Novel RGAG1-BCOR gene fusion revealed in a somatic soft tissue sarcoma with a long follow-up
Source: Virchows Arch. 2021 Jul 31;480(5):1107–14. doi: 10.1007/s00428-021-03160-z (PMC9033707; doi:10.1007/s00428-021-03160-z)

## Slide 1
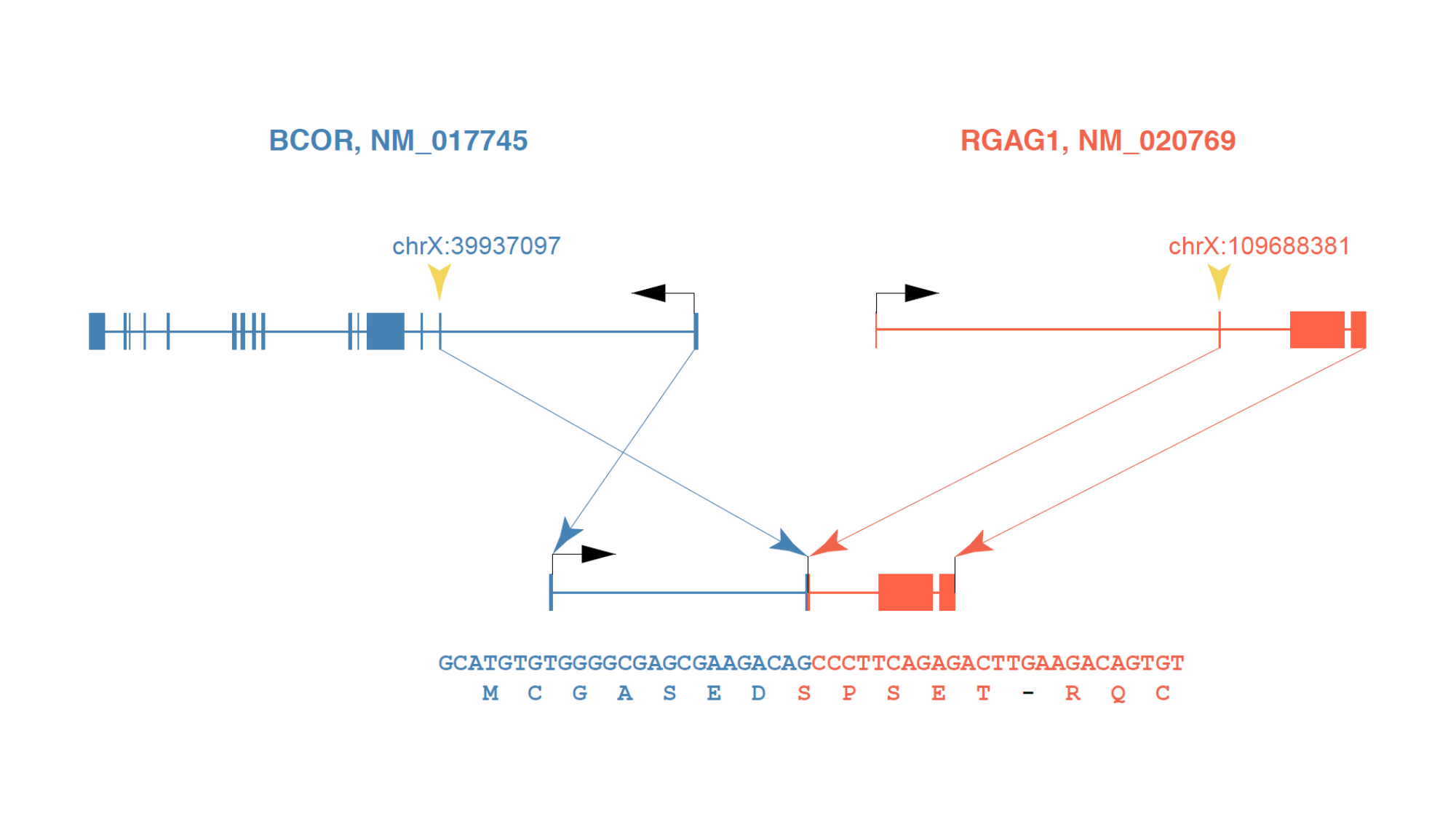

Supplement: Supplementary file 1 — Supplementary file1 (PPTX 109 KB) [file 428_2021_3160_MOESM1_ESM.pptx]

## Slide 1
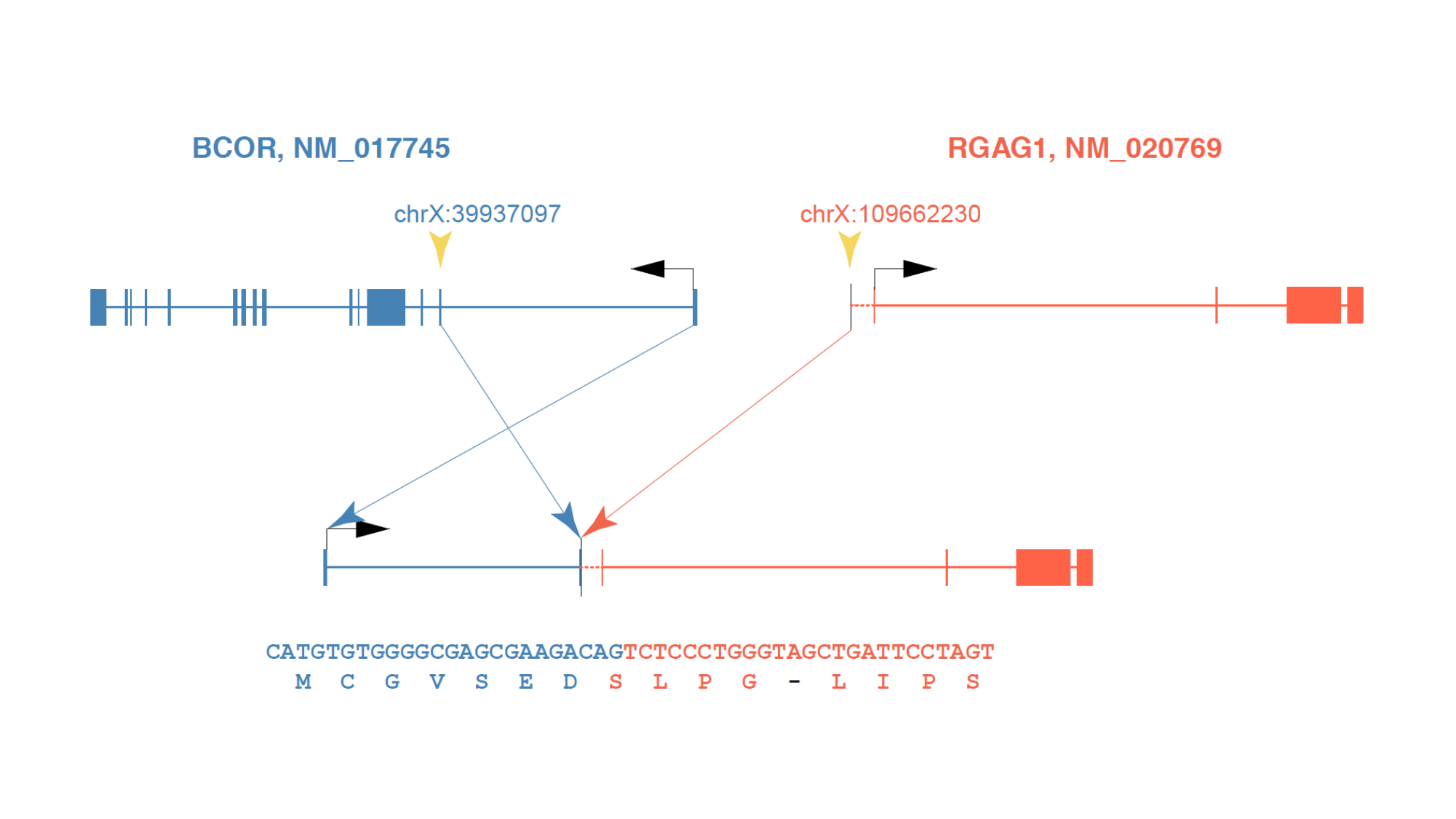

Supplement: Supplementary file 2 — Supplementary file2 (PPTX 101 KB) [file 428_2021_3160_MOESM2_ESM.pptx]

## Slide 1
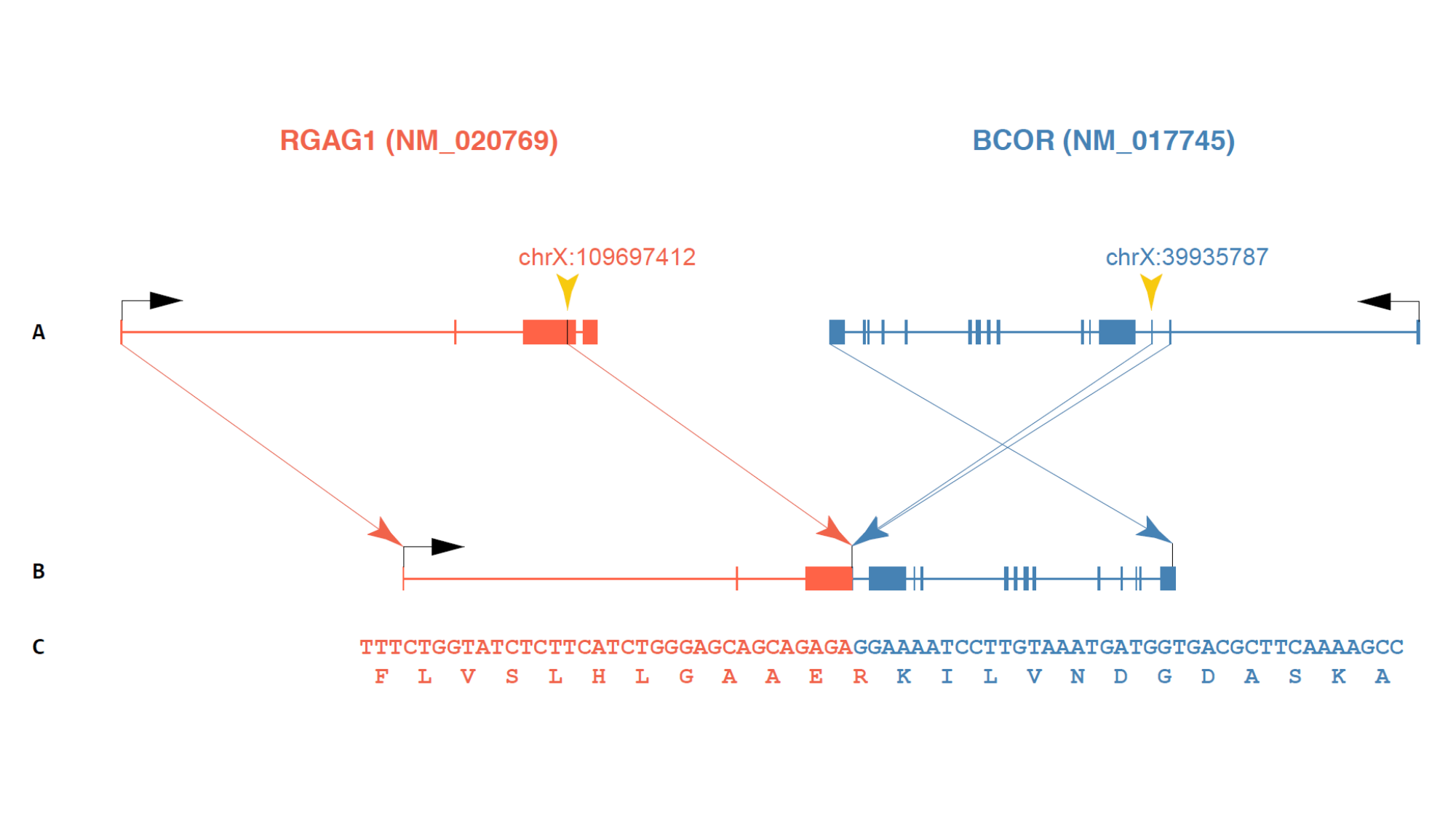

Supplement: Supplementary file 3 — Supplementary file3 (PPTX 118 KB) [file 428_2021_3160_MOESM3_ESM.pptx]
